# Vaginal breech deliveries: Trend in the past 30 years

**DOI:** 10.4274/tjod.47123

**Published:** 2014-12-15

**Authors:** Cihan Çetin, Cansun Demir

**Affiliations:** 1 Çukurova University Faculty of Medicine, Department of Obstetric and Gynecology, Division of Perinatology, Adana, Turkey

**Keywords:** Breech presentation

## Dear Editor,

Breech presentation is encountered in 3-4% of pregnancies at term^(1)^. However, it is more common in preterm deliveries and in pregnancies complicated by myoma uteri, placenta previa and fetal anomalies. On account of this, obstetricians must have the required skills in order to manage these deliveries.

During the past years, there is a decreasing trend in delivering babies vaginally if they are in breech presentation regardless of the its type. There are three main types of breech presentations: Frank breech, complete breech and footling presentations. In case of footling and complete breech presentations, most authors agree to deliver via cesarean section (C/S). However, in cases of frank breech, there is no agreement on the way of delivery.

Especially after the Term Breech Trial published in 2000, many clinicians changed their attitude in managing breech presentations towards C/S^(2)^. That’s because, this study showed that babies delivered via C/S because of breech presentation, had better perinatal mortality, neonatal mortality and serious neonatal morbidity rates than those delivered vaginally. This study and probably the increasing medicolegal issues, caused obstetricians to be more defensive and thus, resulting more liberal indications for C/S. This increasing trend in C/S deliveries is also causing inadequate education and expertise for obstetricians in breech deliveries, which may also increase complications where vaginal breech delivery trial is inevitable. Today, major maneuvers like Mauriceau-Smellie-Veit, Prague, Bracht etc. required for term breech deliveries are seldomly shown to residents. This will probably increase the perinatal morbidity and mortality rates in these cases in the future.

As we aimed to investigate our clinic’s management attitude toward breech presentations in the past 30 years, we also encountered a decreasing trend in vaginal deliveries. First, between 1980-1983 C/S rate for breech presentations were 15%. Later, between 1993-1997 rate elevated to 73% and lastly between 2008-2013, rate was 86%^(3,4)^. Our increasing trend is consistent with other countries^(5,6)^.

Although the lack of support for Term breech trial, there is a globally increasing trend for C/S rates for breech deliveries. Major question is “Do we really decrease the perinatal complications by C/S?” and “How many C/S should be performed in order to prevent one complication related to vaginal birth?”. In our opinion, before the extinction of trained obstetricians in breech delivery, vaginal birth trial should be an alternative for carefully selected patients. By this way, continuity of education can be possible and C/S rates can be lowered.
